# In the heat of connection: using infrared thermal imaging to shed new light into early parent-infant co-regulation patterns

**DOI:** 10.3389/fnbeh.2024.1388886

**Published:** 2024-04-05

**Authors:** Sarah Nazzari, Fatemeh Darvehei, Ellie Nicole Jensen, Samuele Lucchin, Anastasiia Samoukina, Livio Provenzi

**Affiliations:** ^1^Department of Brain and Behavioral Sciences, University of Pavia, Pavia, Italy; ^2^Developmental Psychobiology Lab, IRCCS Mondino Foundation, Pavia, Italy

**Keywords:** infrared thermal imaging, emotion regulation, Autonomic Nervous System, parent-infant interaction, synchrony

## 1 Introduction

Soon in life, humans' bio-behavioral systems are organized and integrated in response to the social environments (Beeghly and Tronick, 2011; Rothbart et al., 2011). Co-regulation between parent and infant, involving the reciprocal coordination of physiology, emotion, and behaviors during the interaction (Butler and Randall, 2013), plays an important role in these organizational processes. Noteworthy, patterns of parent-infant co-regulation across the first year of life influence several emerging developmental processes such as attachment, socio-emotional development and stress regulation (Feldman, 2007b; Evans and Porter, 2009; Kiel et al., 2024). In this vein, delving into the diverse facets of parent-infant co-regulation is increasingly acknowledged as a key focus in developmental science.

The biological stress response systems, including the Autonomic Nervous System (ANS), are thought to play a key role in co-regulation processes (Feldman, 2007a). Small but growing literature suggests that synchronous interactions may modulate mother-infant autonomic physiology such as cardiac activity (Feldman et al., 2011; Porter et al., 2022). Traditional gold standard physiological methods for the assessments of the ANS functioning include electrocardiography or skin conductance (Ioannou et al., 2014). These classical techniques require the use of contact sensors, restrictions of movements, and high levels of compliance of the participants, resulting in more invasive and less ecological ANS measurements. This can be of particular concern with very young populations where the compliance of participants cannot be assured, and all experimental manipulations might result in biased evaluation of infants' emotional state and of the dyadic interaction.

Infrared thermal imaging (ITI) is an increasingly employed technique that enables to accurately measure cutaneous temperature in a non-invasive, ecological, and contactless manner. ITI devices, also known as thermal cameras, can capture the naturally emitted infrared thermal radiation of the human body and convert it into a radiometric thermal image that digitally maps the superficial temperature distribution of the object of measurement. Recent advances in infrared technology led to up-to-date detectors that guarantee high thermal resolution and accuracy, as well as relatively low-cost mobile thermal cameras options. While ITI use in biomedical fields is growing, there are still sparse applications of this technique to developmental science and particularly, to the assessment of parent-infant interaction. In this opinion, we describe the nature of the thermal response and how it can be molded in real time by social interaction, we review available evidence of ITI as applied to parent-infant interaction, and we outline potentialities and future direction for ITI as a new promising avenue for the accurate and non-invasive investigation of early parent-infant co-regulation.

## 2 The affective thermal response

ITI techniques allow to examine variations in the ANS activity reflected by cutaneous temperature modulations. Several parameters of the ANS activity exhibit a specific thermal signature (Cardone and Merla, 2017) and can be estimated at a distance employing bioheat transfer models. Thermal directional changes on the skin related to blood flow are the most widely explored thermal indices (Ioannou et al., 2014). Blood flow transfers the heat from the body core to the skin and is regulated by vascular processes under the control of the sympathetic (SNS) and parasympathetic (PNS) nervous systems. Vasoconstriction is mainly controlled by the SNS and induces a local decrease in skin temperature. In contrast, vasodilation has been associated with an increase in cutaneous surface thermal radiation which depends on a greater cutaneous blood perfusion. In a seminal study Kistler et al. (1998) used laser Doppler flowmetry together with ITI to measure, respectively, blood flow and temperature of the fingertip while exposing 30 participants to various stressors like horror movies and acupuncture. They reported a decrease in blood flow (i.e., vasoconstriction), followed by a 15-s delay in fingertip temperature decrease, with over 92% of occasions where the blood flow decrease led to fingertip cooling, suggesting thermal imaging reflects sympathetic activity. Since then, several studies have demonstrated the reliability of ITI as a measure of autonomic activation, by comparing it with simultaneous recordings made with gold-standard methods, including ECG and skin conductance (e.g., Shastri et al., 2009; Kuraoka and Nakamura, 2011). These studies suggest that ITI can reliably capture psychophysiological arousal states while differentiating them from baseline conditions (Shastri et al., 2009; Nhan and Chau, 2010). Furthermore, research has shown that ITI and skin conductance have similar detection power both in human and non-human primates (Coli et al., 2007; Shastri et al., 2009; Kuraoka and Nakamura, 2011; Pavlidis et al., 2012), but different latencies (Merla and Romani, 2007; Kuraoka and Nakamura, 2011). Specifically, a key difference in the onset of the responses was observed, with temperature variations being detected at least 10 s after a stimulus as compared to skin conductance being able to show changes in signal much faster, within 3 s (Merla and Romani, 2007; Kuraoka and Nakamura, 2011).

Studies that investigated the ANS response during social interaction through ITI have typically been focused on measuring facial cutaneous thermal variations both from a temporal and topographic perspective (Ioannou et al., 2014). The face is particularly relevant because it is not obscured, can be easily recorded and is highly involved in social interaction. Facial temperature is related to fluctuations in the distributions of blood in the vessels and is regulated by SNS and PNS activity, being responsive to emotional stimuli in the environment (Kreibig, 2010). As previously mentioned, vasodilation is related to an increase in facial temperature, whereas vasoconstriction implies a decrease in facial temperature. These changes in the infrared emissivity of the facial skin can be captured through ITI and can be monitored over time and across different regions of the face by using simple metrics such as the thermal difference between two time points or two spatial regions. When it comes to the latter, regions of interest (ROIs) within the face are usually employed, with the most studied ROIs being the nose or the nasal tip, the periorbital or supraorbital area, the forehead, the orbicularis oculi, and the maxillary area (Ioannou et al., 2014). In particular, the nasal tip shows the most consistent thermal response to emotional/stressful stimuli with a decrease of the temperature of this ROIs being consistently found in response to emotional and stressful stimuli and being related to sympathetic adrenergic vasoconstrictor activity (Merla and Romani, 2007; Ebisch et al., 2012).

## 3 The facial thermal response to parent-infant interactions

Thanks to its non-invasiveness, ITI offers the possibility to study humans' emotional and physiological autonomic reactions during social interactions in a more naturalistic setting. Several studies have been conducted on the modulation of facial temperature when interacting with one or more persons in adults (e.g., Hahn et al., 2012; Park et al., 2013; Ponsi et al., 2019). In contrast, only few human studies employed ITI to assess some aspects of parent-infant interactions (Ebisch et al., 2012; Manini et al., 2013; Aureli et al., 2015; Ioannou et al., 2021). Albeit preliminary, evidence indicated that infants' facial temperature is responsive to what happens in the social environment. In particular, Aureli et al. (2015) reported an increase in infants' temperature of the nasal tip and forehead during a face-to-face mother-infant interaction at 3–4 months of age, possibly suggesting a PNS activation during the experimental paradigm. Furthermore, thermal variations were related to infants' behavior, with greater increase in infant's temperature being related to greater engagement with the environment during the still face episode and less negative affectivity. This might suggest that infants' PNS activation might support infants' social engagement with the surrounding environment, as predicted by the Polyvagal Theory (Porges, 2001). Likewise, an increase in 2-month-old infants' facial temperature while interacting with a stranger, as compared to the mother, was reported in another study (Ioannou et al., 2021), being suggestive of a greater involvement of the PNS as social challenges increase. This was paralleled by behavioral data showing greater gaze duration to the stranger as compared to the mother. Although replication of these findings in larger samples is needed, this preliminary evidence indicates that infants' thermal variations during social interaction might be a sensitive marker of PNS activation. Additionally, an activation of the SNS, as indicated by a decrease of the temperature of infants' maxillary area and nasal tip was detected in older children (38–42 months) through ITI during more stressful paradigm, such as the “mishap” paradigm (Ebisch et al., 2012). This paradigm consisted of five episodes, including a “mishap” episode, during which the child is left alone by the experimenter and accidentally “broke” the toy that was given to him. This is followed by the return of the experimenter that did not speak for 30 s and looked at the broken toy and by the “soothing” of the child (Cole et al., 1992). A similar facial thermal modulation was reported in mothers observing their children (Ebisch et al., 2012) and in unrelated women watching unknown children (Manini et al., 2013) during the “mishap” paradigm. Most interestingly, a positive association between maternal and child thermal response during the stressful task was reported (Ebisch et al., 2012) and autonomic attunement was modulated by the degree of relatedness, with a slightly stronger and much faster attunement in thermal responses of mother-child dyads as compared to unknown women-child dyads, as showed by cross-correlation analysis (Manini et al., 2013). This initial evidence suggests that the assessment of mother-infant facial thermal variations might provide further insight into the phenomenon of physiological co-regulation. Further studies using ITI are needed to investigate the degree of physiological synchrony in facial thermal variations among mothers and infants in response to both stressful and non-stressful situations, as well as their role in the development of infant stress regulation.

## 4 Discussion

Paralleling the rising interest into the mechanisms underlying the impact of early interactions in human development, there is an urgent need of sensitive and ecologically valid methods that allow to evaluate infants' physiology during real interactions in a naturalistic environment. Traditional physiological parameters, such as heart rate or skin conductance, are now very simple to be collected and analyzed and are increasingly employed in pediatric populations (e.g., Provenzi et al., 2015; Nava et al., 2016; Quadrelli et al., 2019; Reali et al., 2021; Nazzari et al., 2022a). Limitations of these methods include that they are mostly obtained through contact sensors, required participants' compliance to correctly wear the device and a certain amount of time for their accurate application. These drawbacks become particularly relevant when testing challenging populations, such as infants or clinical samples. In the current opinion, we suggest the potential of ITI as a useful tool to investigate parent-infant autonomic co-regulation in the context of early interactions. Thanks to its non-invasiveness and contact-free nature, ITI provides the unique opportunity to study natural social interactions without interfering with spontaneous behaviors in an ecological valid setting. This can be especially important when it comes to the investigation of social interactions early in life or in especially vulnerable samples. For example, ITI might be a useful option to study co-regulation processes in samples of atypically developing children with high skin sensitivity, such as children with autism spectrum disorder. Likewise, children with somatic diseases, such as atopic dermatitis, or conditions related to skin fragility, such as very small premature babies, might benefit for the use of a contact-free techniques, such as ITI, that does not involve the application of electrodes or other devices on their skin.

Available studies showed that, as early as 2 months of age, facial thermal variations are sensitive to social challenges and can represent a marker of autonomic activation (Ebisch et al., 2012; Aureli et al., 2015). It is now widely acknowledged that a complex interplay among genetic and environmental factors is involved in shaping infants' individual differences in stress regulation (Grant et al., 2009; Nazzari et al., 2022b). Thus, future research into the role of individual or caregiving-related influences on early infant thermal regulation is required.

**Figure 1:**
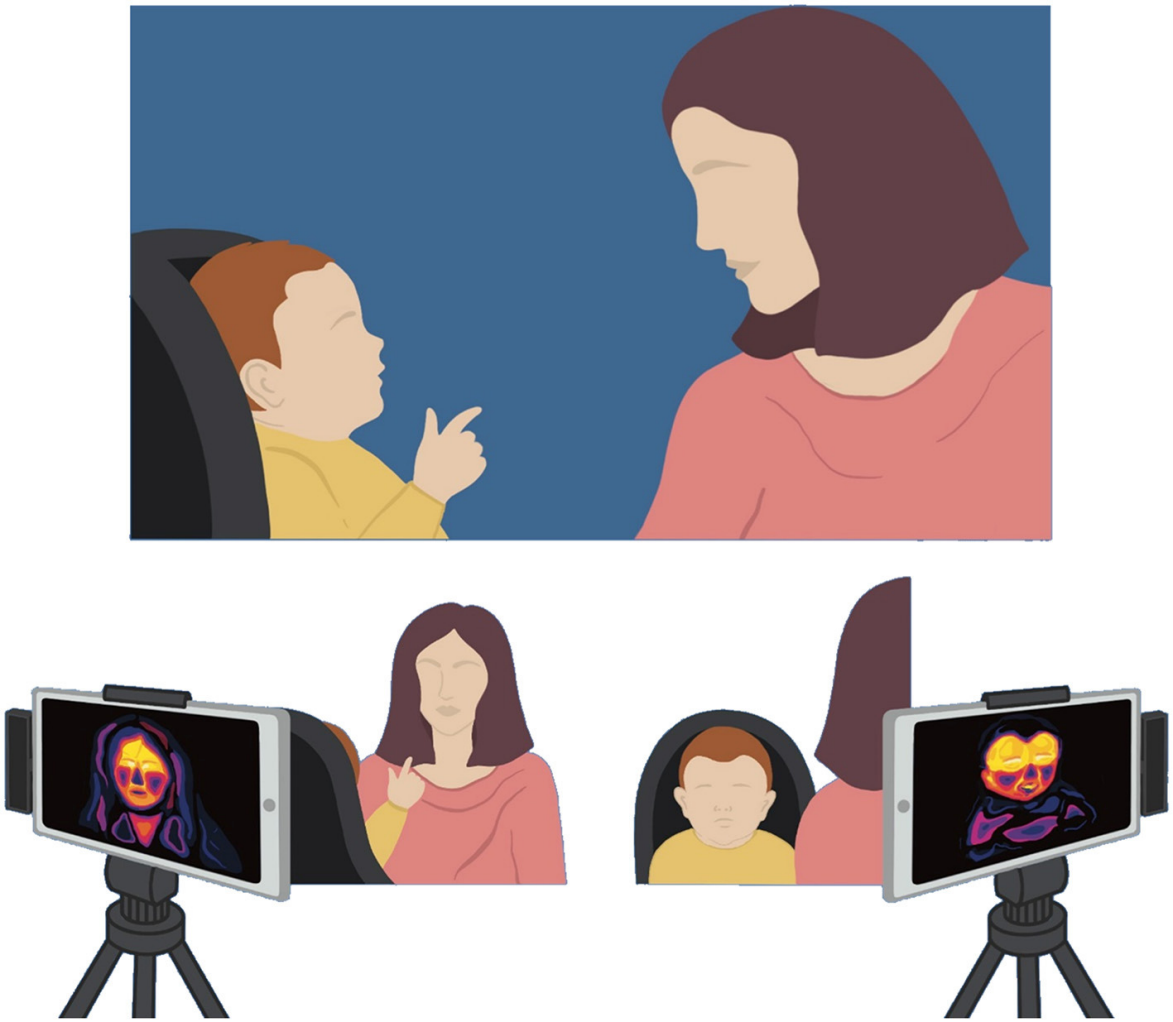
Schematic representation of an illustrative setting for the assessment of mother-infant thermal regulation through ITI.

As promising as ITI appears to be, it is equally true that several methodological issues deserve considerations when designing an ITI study. First of all, it is important to keep in mind that despite the advantages offered by ITI, the thermal signal that result from vascular changes is rather slow and has a longer latency as compared to traditional methods such as skin conductance (Kuraoka and Nakamura, 2011). Furthermore, individual and environmental sources of interference with thermal assessment need to be accounted for, as well as technical issues involving distance, motion and angles. An illustrative setting for the assessment of mother-infant thermal regulation through ITI is graphically schematized in Figure 1. We recommend consulting the International Academy of Thermology (IACT) guidelines (Thermography Guidelines, 2002), for a broader discussion. Specific considerations might concern studies on pediatric samples. For example, maternal touching of the face of the child during the ITI recordings might confound the assessment and should thus be avoided. Likewise, a manual tracking system might be recommended to avoid noise related to infants' rapid head movements. Lastly, studies that correlate thermal measures with other well-established physiological measures of stress regulation early in life are needed.

In conclusion, ITI represents a potential versatile and reliable tool for the investigation of physiological responses to social exchanges early in life in both typical and atypical populations which still warrants further explorations. The potential contribution of ITI extends to the examination of early patterns of bio-behavioral synchrony as they unfold in a more ecological and naturalistic setting. Furthermore, the development of small, mobile, low-cost thermal device imaging device holds the promise to further bridge the gap between a constrained laboratory setting and a natural real-life scenario. This opens the door for a more inclusive participation of diverse populations in developmental research. Recognizing that early co-regulation of physiology and behavior between parent and infant lay the ground for later human social interactions and children wellbeing and adaptation (Feldman, 2007a), the use of ITI stands to enrich our understanding of these fundamental processes.

## Author contributions

SN: Conceptualization, Supervision, Writing – original draft, Writing – review & editing. FD: Investigation, Writing – original draft. EJ: Writing – original draft. SL: Investigation, Writing – original draft. AS: Visualization, Writing – original draft. LP: Supervision, Writing – review & editing.
